# Supplementary material for: Detecting alternative attractors in ecosystem dynamics
Source: Commun Biol. 2021 Aug 17;4:975. doi: 10.1038/s42003-021-02471-w (PMC8370982; doi:10.1038/s42003-021-02471-w)
Supplement: Supplementary file 2 — Supplementary information [file 42003_2021_2471_MOESM2_ESM.pdf]

Supplementary Information

Detecting alternative attractors in ecosystem dynamics

Torbjörn Säterberg[1*], Kevin McCann[2]

[1] Swedish University of Agricultural Sciences, Department of Aquatic Resources, Skolgatan 6, SE-742 42 Öregrund, Sweden.

[2] Department of Integrative Biology, University of Guelp, Guelph, Ontario, Canada N1G 2W1.

*Correspondence to: torbjorn.saterberg@slu.se.

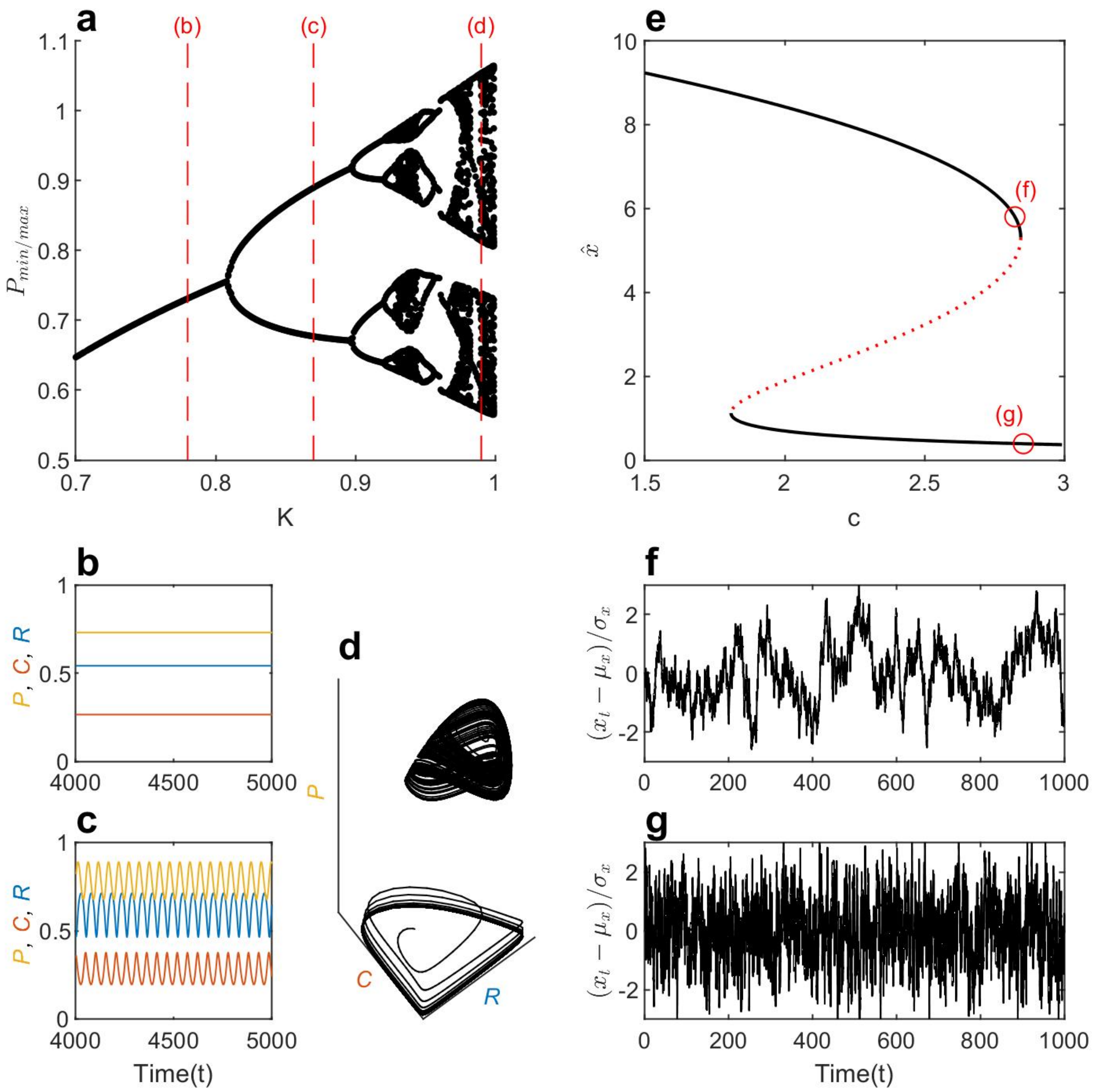

Supplementary Figure 1. Alternative dynamical regimes predicted by a non-linear model and a stochastic model. **a**, Bifurcation diagram of a food-chain model (eq. 7; See Methods). Maximum and minimum predator densities (*P*) are here predicted for different resource carrying capacities *K*. Specific values of the resource carrying capacity *K* can, for example, give rise to: **b**, Equilibrium dynamics, **c**, 2-point limit cycles, and **d**, alternative dynamical regimes (depending on initial conditions either: (i) chaotic dynamics or (ii) a 2-point limit cycle of the resources (*R*) and consumer (*C*) with the predator (*P*) going extinct). **e**, Bifurcation diagram for a one-dimensional alternative stable state model[S1-S3] (eq. 9; See Methods). Alternative equilibria ($\hat{\boldsymbol{x}}$) are here predicted for different harvest rates (*c*). Black lines represent stable equilibria and red dotted line represent unstable equilibria. When exposed to stochasticity the model produces alternative stochastic regimes, characterized by different temporal time series signals, depending on in which basin of attraction the model state resides (**f** vs. **g**).

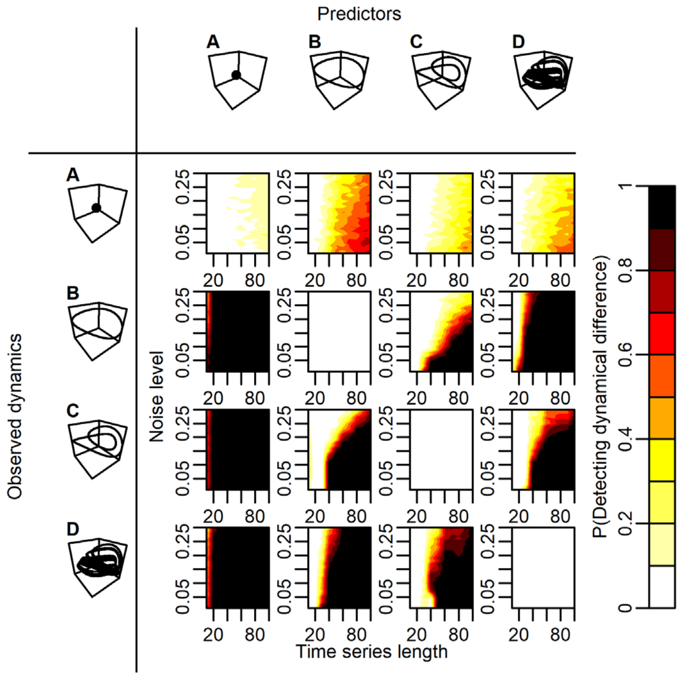

Supplementary Figure 2. Detecting alternative dynamical attractors in systems dominated by internally-driven signals. The figure shows the probability of detecting significant difference (P(Detecting dynamical difference); color bar) in absolute prediction error for across ($|\widehat{\boldsymbol{Y}}_{\boldsymbol{i}}(t)|\boldsymbol{M}_{\boldsymbol{j}} - \boldsymbol{Y}_{\boldsymbol{i}}(\mathrm{t})|$) and within regime predictions ($|\widehat{\boldsymbol{Y}}_{\boldsymbol{i}}(t)|\boldsymbol{M}_{\boldsymbol{i}} - \boldsymbol{Y}_{\boldsymbol{i}}(\mathrm{t})|$) in data produced by a food-chain model. The observed dynamical regimes, $\boldsymbol{Y}_{\boldsymbol{i}}(t)$, which are predicted using within ($\widehat{\boldsymbol{Y}}_{\boldsymbol{i}}(t)|\boldsymbol{M}_{\boldsymbol{i}}$) and across regime dynamics ($\widehat{\boldsymbol{Y}}_{\boldsymbol{i}}(t)|\boldsymbol{M}_{\boldsymbol{j}}$) are shown in the first column: A, equilibrium with additive observation noise; B, a 2-point limit cycle; C, a 4-point limit cycle; D, a chaotic attractor. Across regime predictors, $\boldsymbol{M}_{\boldsymbol{j}}$, are displayed in the top row. Probabilities are derived by testing the null-hypothesis H0:$|\widehat{\boldsymbol{Y}}_{\boldsymbol{i}}|\boldsymbol{M}_{\boldsymbol{i}} - \boldsymbol{Y}_{\boldsymbol{i}}| > |\widehat{\boldsymbol{Y}}_{\boldsymbol{i}}|\boldsymbol{M}_{\boldsymbol{j}} - \boldsymbol{Y}_{\boldsymbol{i}}|$ (permutation test; p=0.05) across 100 replicates of each combination of time series length and observation noise level. The consumer in the Predator-Consumer-Resource model (eq. 7) is here used to predict the other two species' dynamics (See Methods).

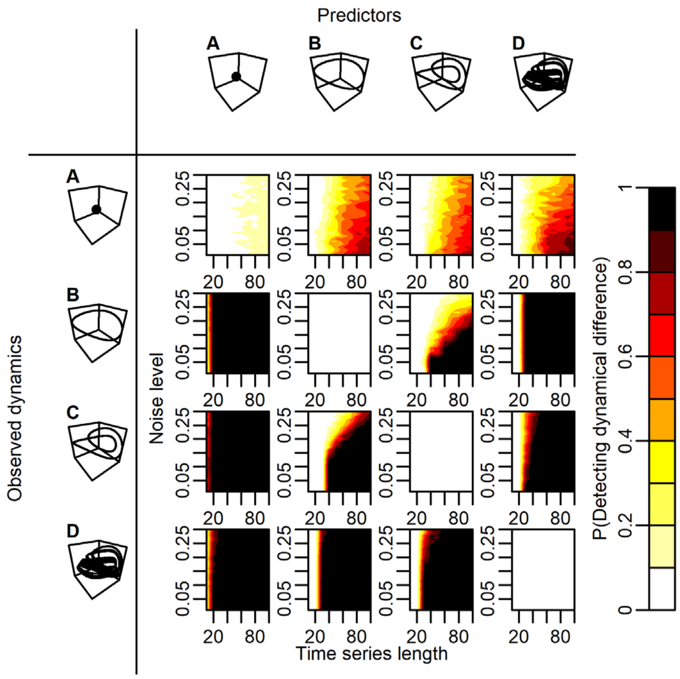

Supplementary Figure 3. Detecting alternative dynamical attractors in systems dominated by internally-driven signals. The figure shows the probability of detecting significant difference (P(Detecting dynamical difference); color bar) in absolute prediction error for across ($|\widehat{\boldsymbol{Y}}_i(t)|\boldsymbol{M}_j - \boldsymbol{Y}_i(\mathrm{t})|$) and within regime predictions ($|\widehat{\boldsymbol{Y}}_i(t)|\boldsymbol{M}_i - \boldsymbol{Y}_i(\mathrm{t})|$) in data produced by a food-chain model. The observed dynamical regimes, $\boldsymbol{Y}_i(t)$, which are predicted using within ($\widehat{\boldsymbol{Y}}_i(t)|\boldsymbol{M}_i$) and across regime dynamics ($\widehat{\boldsymbol{Y}}_i(t)|\boldsymbol{M}_j$) are shown in the first column: A, equilibrium with additive observation noise; B, a 2-point limit cycle; C, a 4-point limit cycle; D, a chaotic attractor. Across regime predictors, $\boldsymbol{M}_j$, are displayed in the top row. Probabilities are derived by testing the null-hypothesis H0:$|\widehat{\boldsymbol{Y}}_i|\boldsymbol{M}_i - \boldsymbol{Y}_i| > |\widehat{\boldsymbol{Y}}_i|\boldsymbol{M}_j - \boldsymbol{Y}_i|$ (permutation test; p=0.05) across 100 replicates of each combination of time series length and observation noise level. The resource in the Predator-Consumer-Resource model (eq. 7) is here used to predict the other two species' dynamics (See Methods).

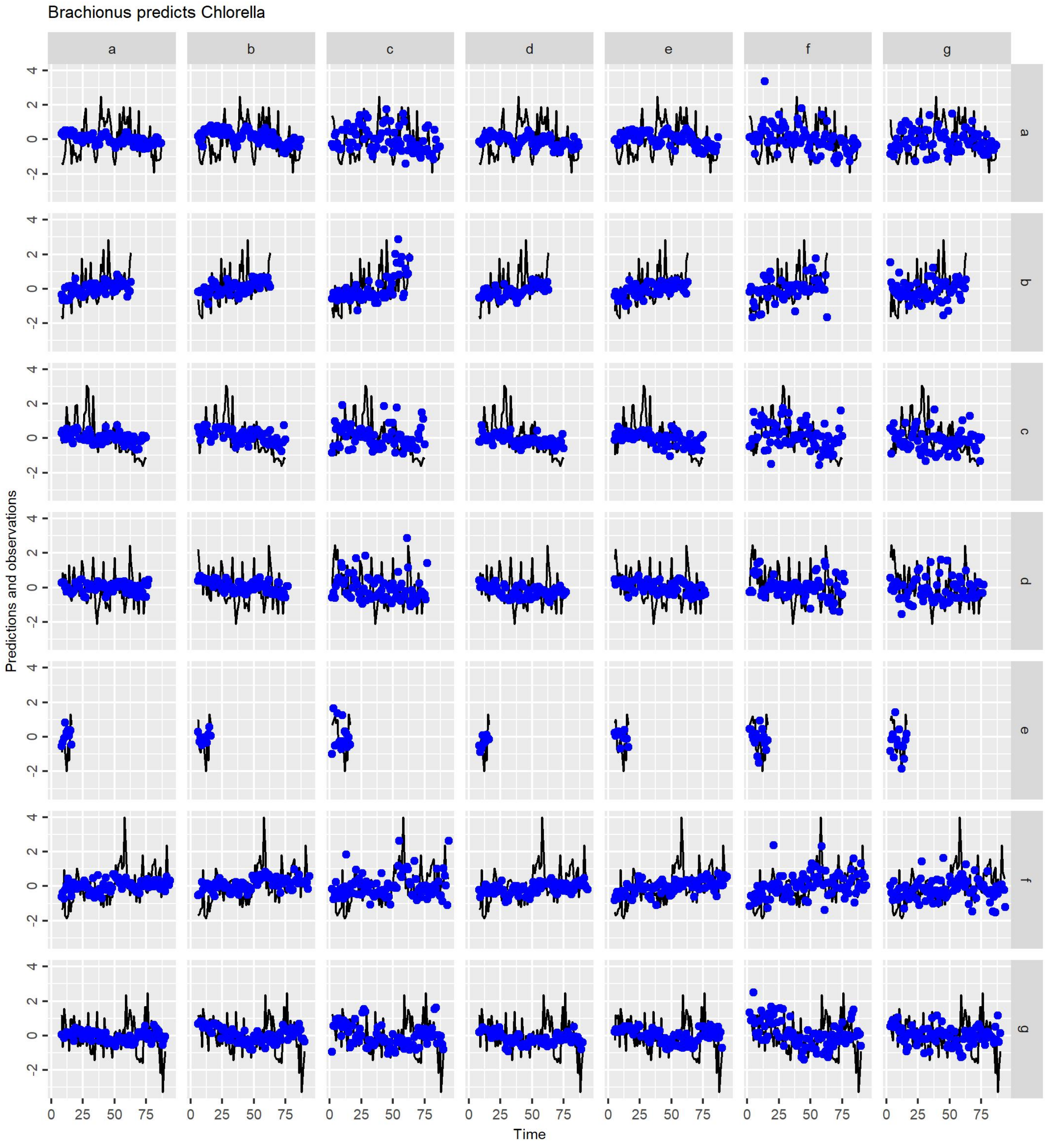

Supplementary Figure 4. ***Chlorella vulgaris*** **"Equilibrium"-time series predicted using "Equilibrium" time series of** ***Brachionus calyciflorus*****.** Observations are displayed as black lines and predictions are shown as blue points. Numbers in grey boxes display indexes of the time series used in Fig. 3. "Equilibrium" time series (a-g; columns) are here used to predict other "Equilibrium" time series (a-g; rows). All time series are standardized (μ=0; sd=1) prior prediction.

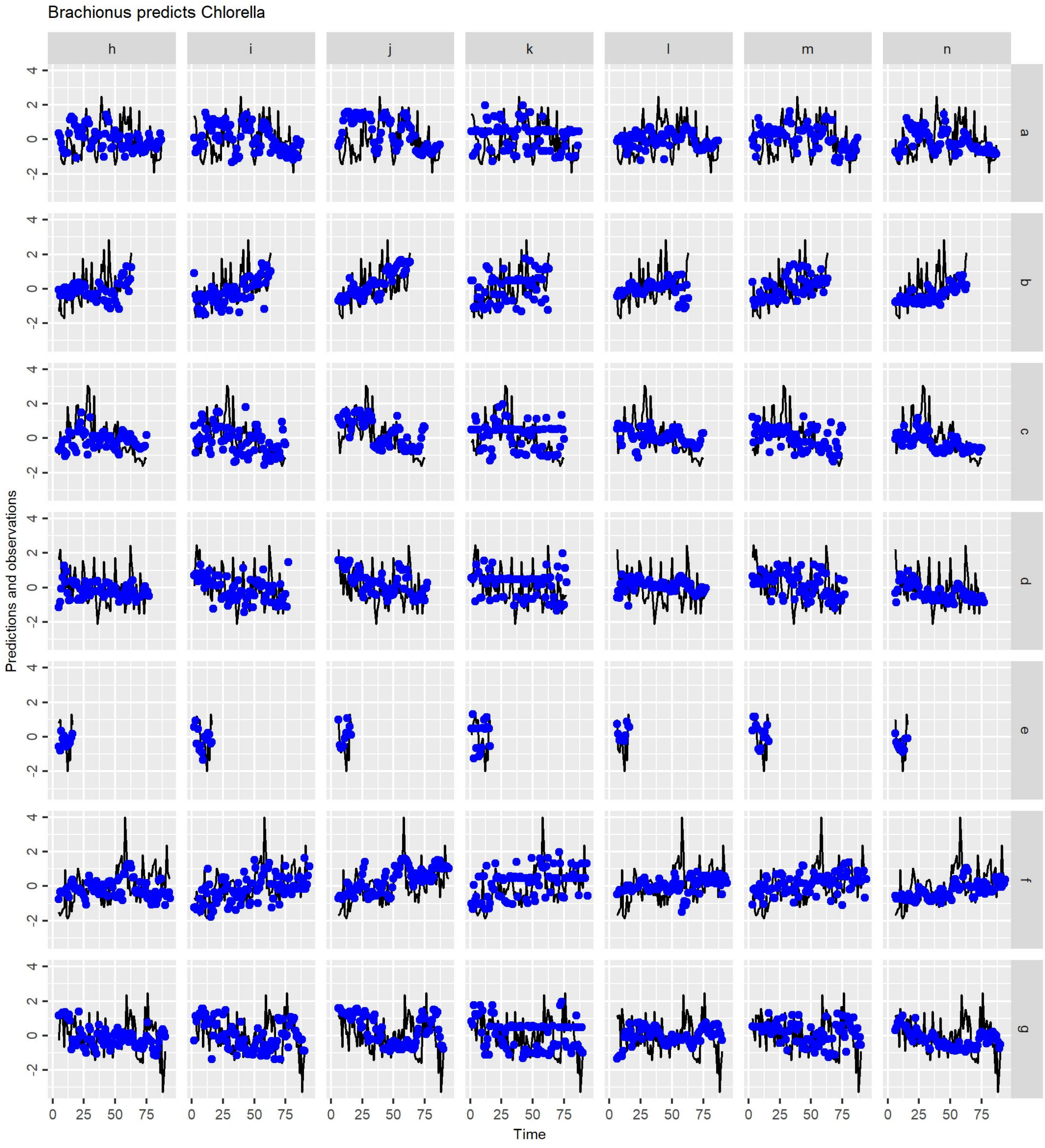

Supplementary Figure 5. ***Chlorella vulgaris*** **"Equilibrium"-time series predicted using "Cyclic" time series of** ***Brachionus calyciflorus*****.** Observations are displayed as black lines and predictions are shown as blue points. Numbers in grey boxes display indexes of the time series used in Fig. 3. "Cyclic" time series (h-n; columns) are here used to predict "Equilibrium" time series (a-g; rows). All time series are standardized (μ=0; sd=1) prior prediction.

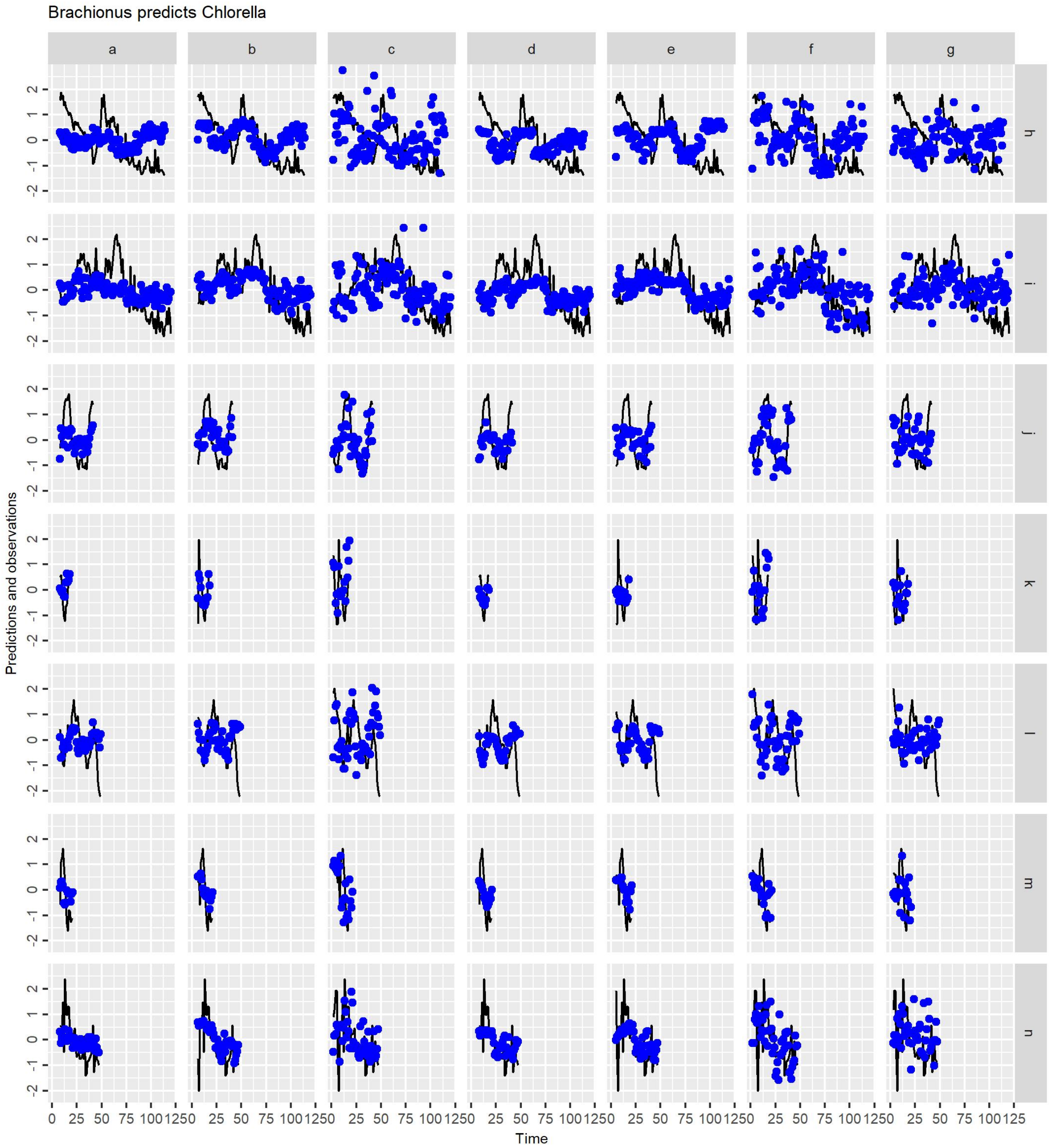

Supplementary Figure 6. ***Chlorella vulgaris*** **"Cyclic"-time series predicted using "Equilibrium" time series of** ***Brachionus calyciflorus*****.** Observations are displayed as black lines and predictions are shown as blue points. Numbers in grey boxes display indexes of the time series used in Fig. 3. "Equilibrium" time series (a-g; columns) are here used to predict "Cyclic" time series (h-n; rows). All time series are standardized (μ=0; sd=1) prior prediction.

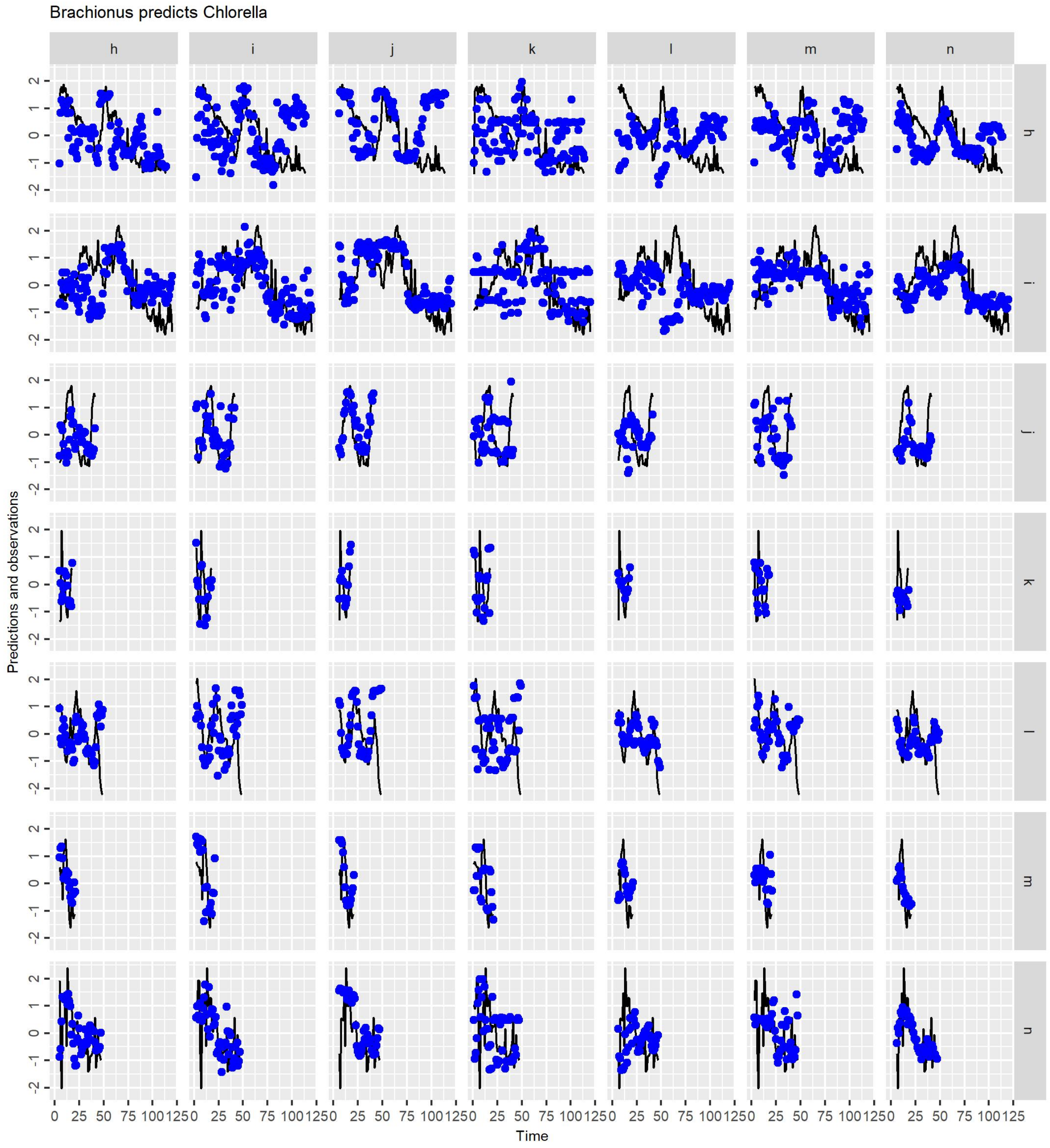

Supplementary Figure 7. ***Chlorella vulgaris*** **"Cyclic"-time series predicted using "Cyclic" time series of** ***Brachionus calyciflorus*****.** Observations are displayed as black lines and predictions are shown as blue points. Numbers in grey boxes display indexes of the time series used in Fig. 3. "Cyclic" time series (h-n; columns) are here used to predict other "Cyclic" time series (h-n; rows). All time series are standardized (μ=0; sd=1) prior prediction.

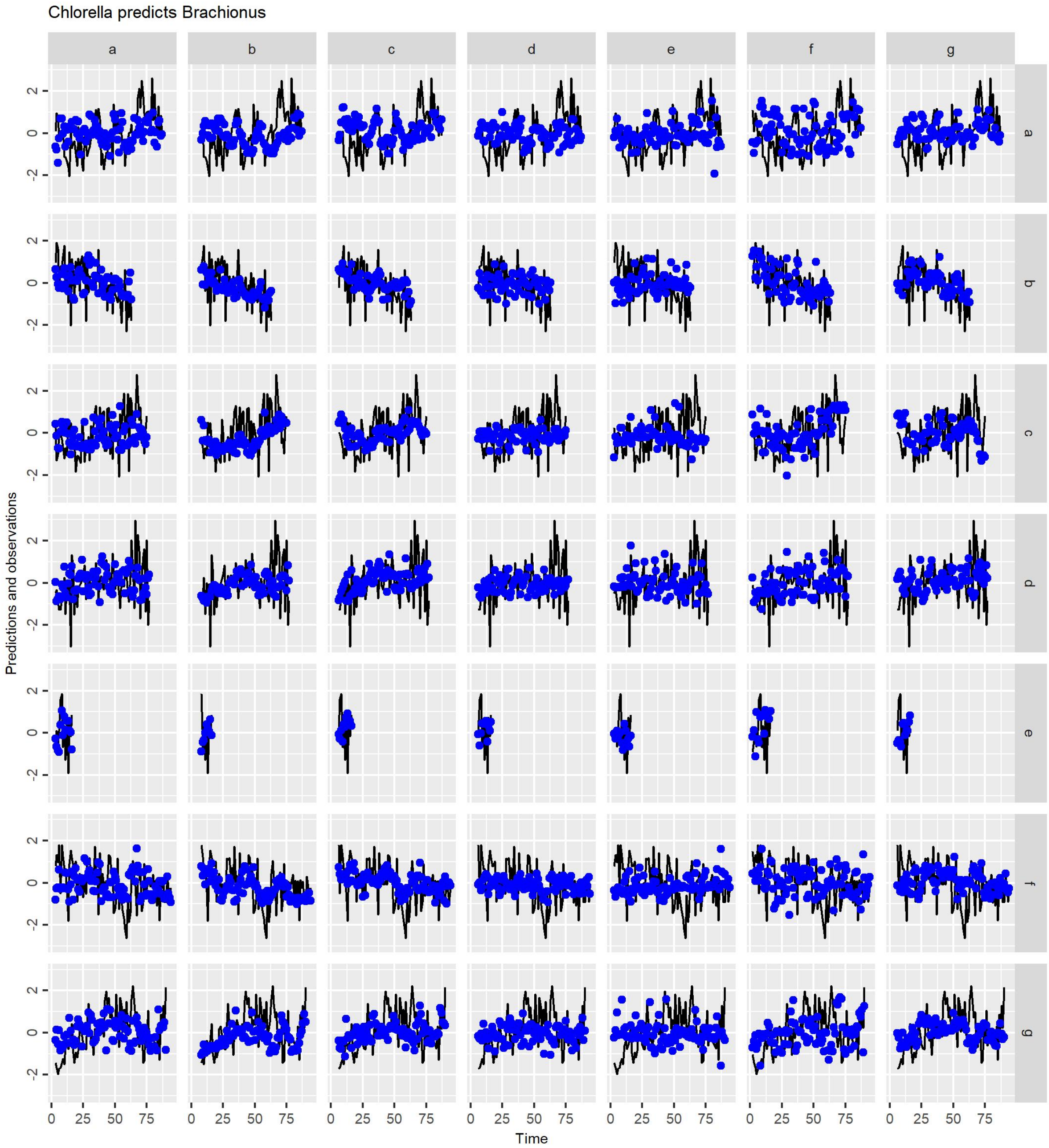

Supplementary Figure 8. ***Brachionus calyciflorus*** **"Equilibrium"-time series predicted using "Equilibrium" time series of *Chlorella vulgaris*.** Observations are displayed as black lines and predictions are shown as blue points. Numbers in grey boxes display indexes of the time series used in Fig. 3. "Equilibrium" time series (a-g; columns) are here used to predict other "Equilibrium" time series (a-g; rows). All time series are standardized (μ=0; sd=1) prior prediction.

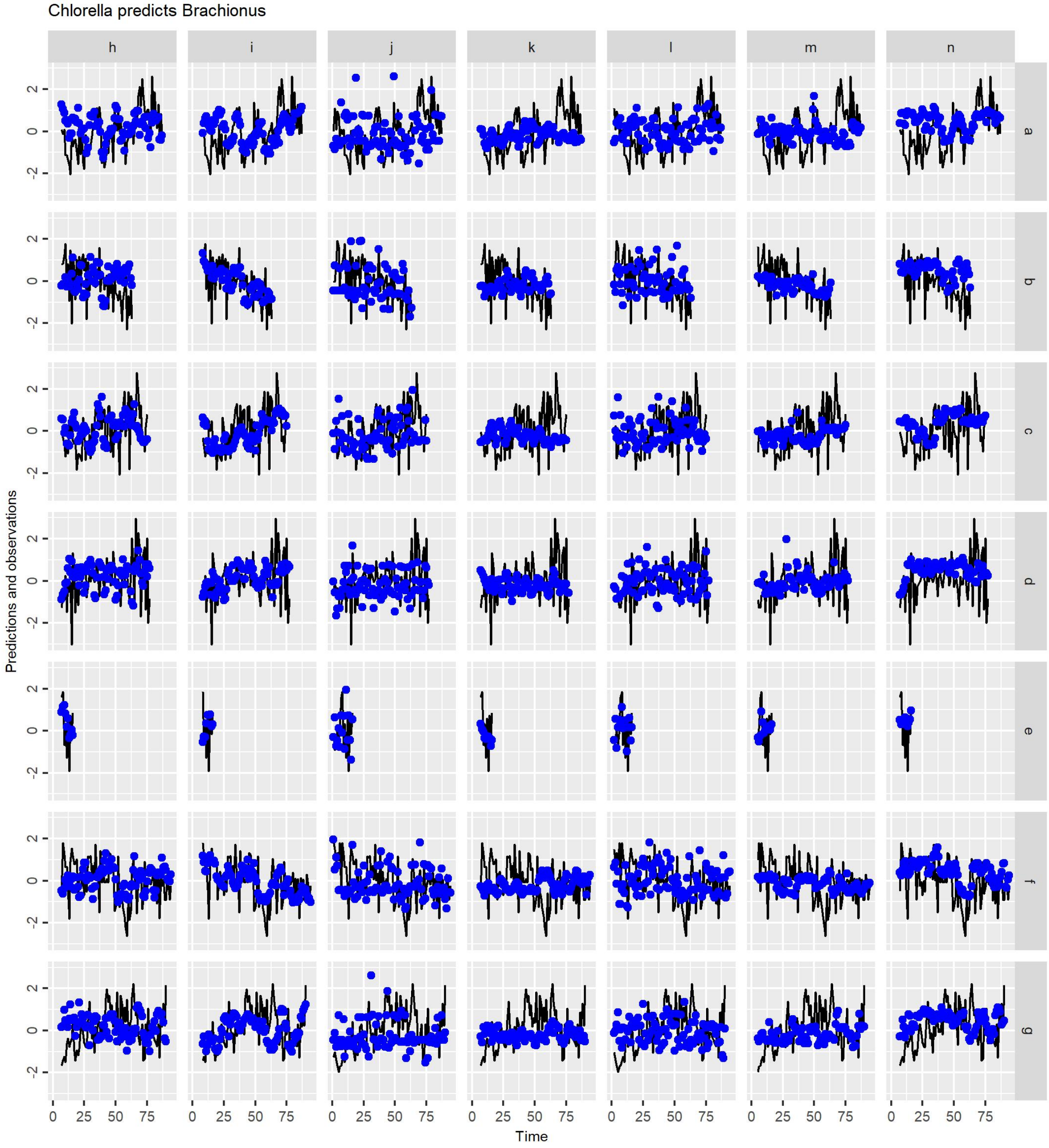

Supplementary Figure 9. ***Brachionus calyciflorus*** **"Equilibrium"-time series predicted using "Cyclic" time series of *Chlorella vulgaris*.** Observations are displayed as black lines and predictions are shown as blue points. Numbers in grey boxes display indexes of the time series used in Fig. 3. "Cyclic" time series (h-n; columns) are here used to predict "Equilibrium" time series (a-g; rows). All time series are standardized (μ=0; sd=1) prior prediction.

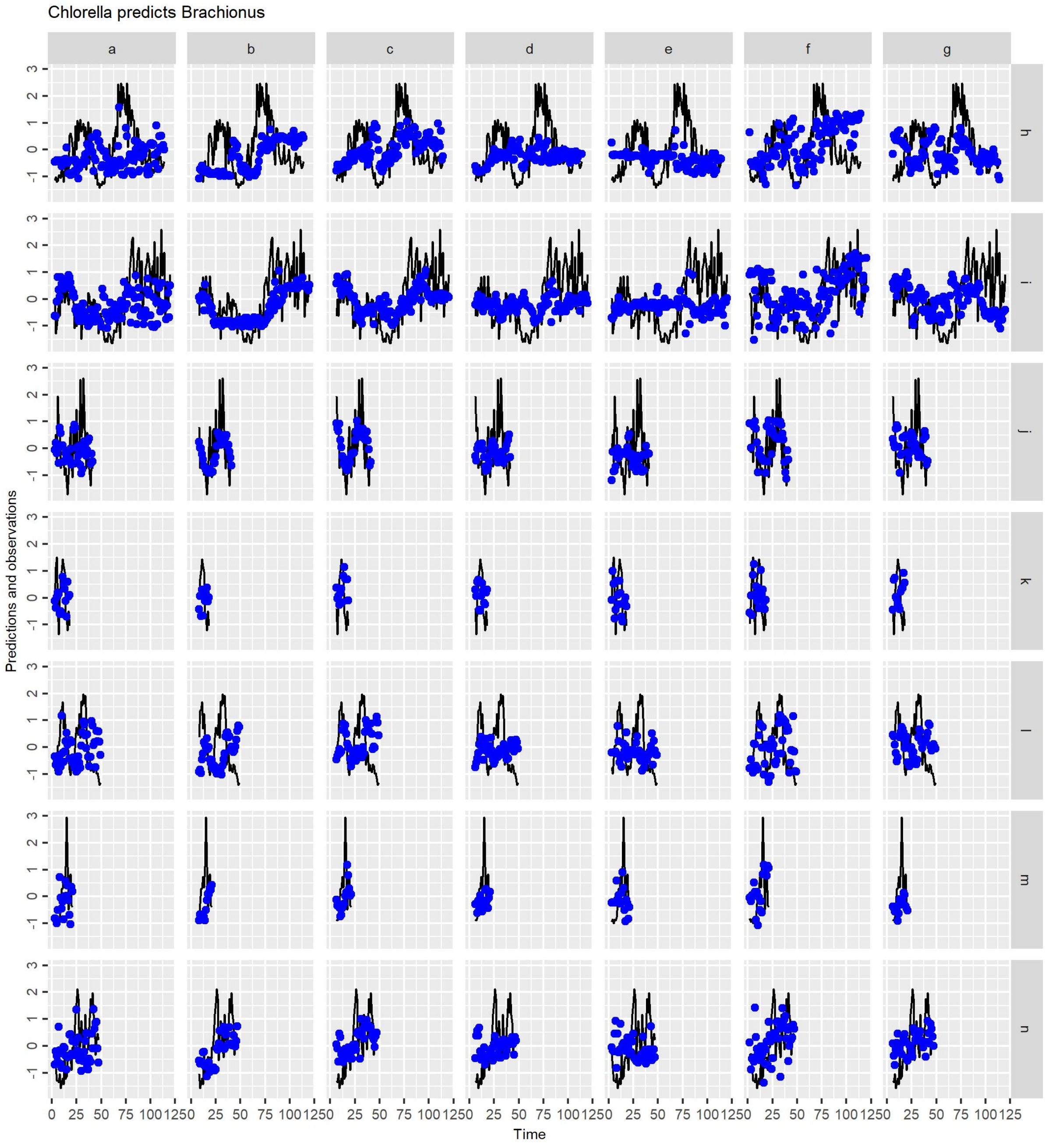

Supplementary Figure 10. ***Brachionus calyciflorus*** **"Cyclic"-time series predicted using "Equilibrium" time series of *Chlorella vulgaris*.** Observations are displayed as black lines and predictions are shown as blue points. Numbers in grey boxes display indexes of the time series used in Fig. 3. "Equilibrium" time series (a-g; columns) are here used to predict "Cyclic" time series (h-n; rows). All time series are standardized (μ=0; sd=1) prior prediction.

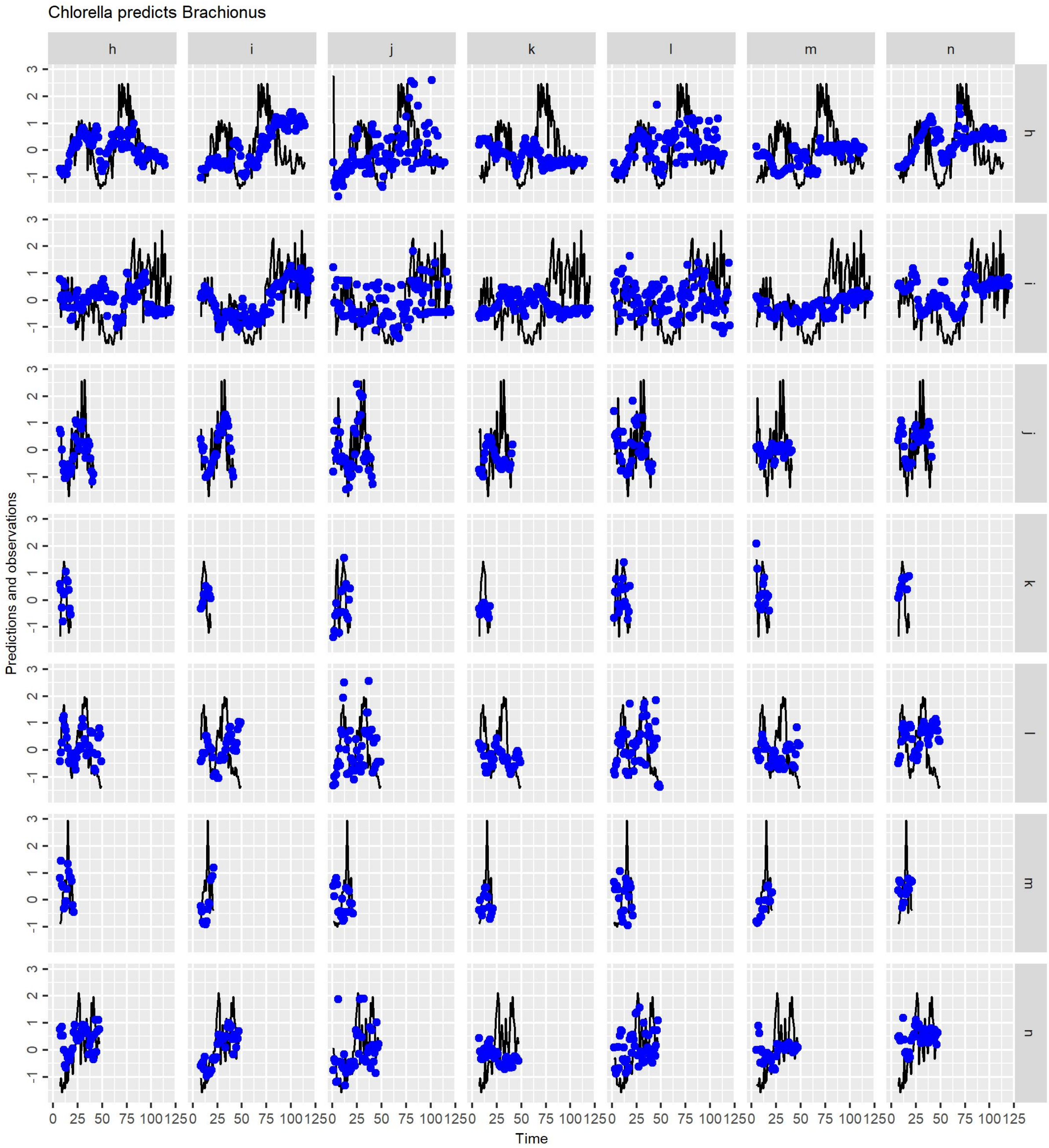

Supplementary Figure 11. ***Brachionus calyciflorus*** **"Cyclic"-time series predicted using "Cyclic" time series of *Chlorella vulgaris*.** Observations are displayed as black lines and predictions are shown as blue points. Numbers in grey boxes display indexes of the time series used in Fig. 3. "Cyclic" time series (h-n; columns) are here used to predict other "Cyclic" time series (h-n; rows). All time series are standardized (μ=0; sd=1) prior prediction.

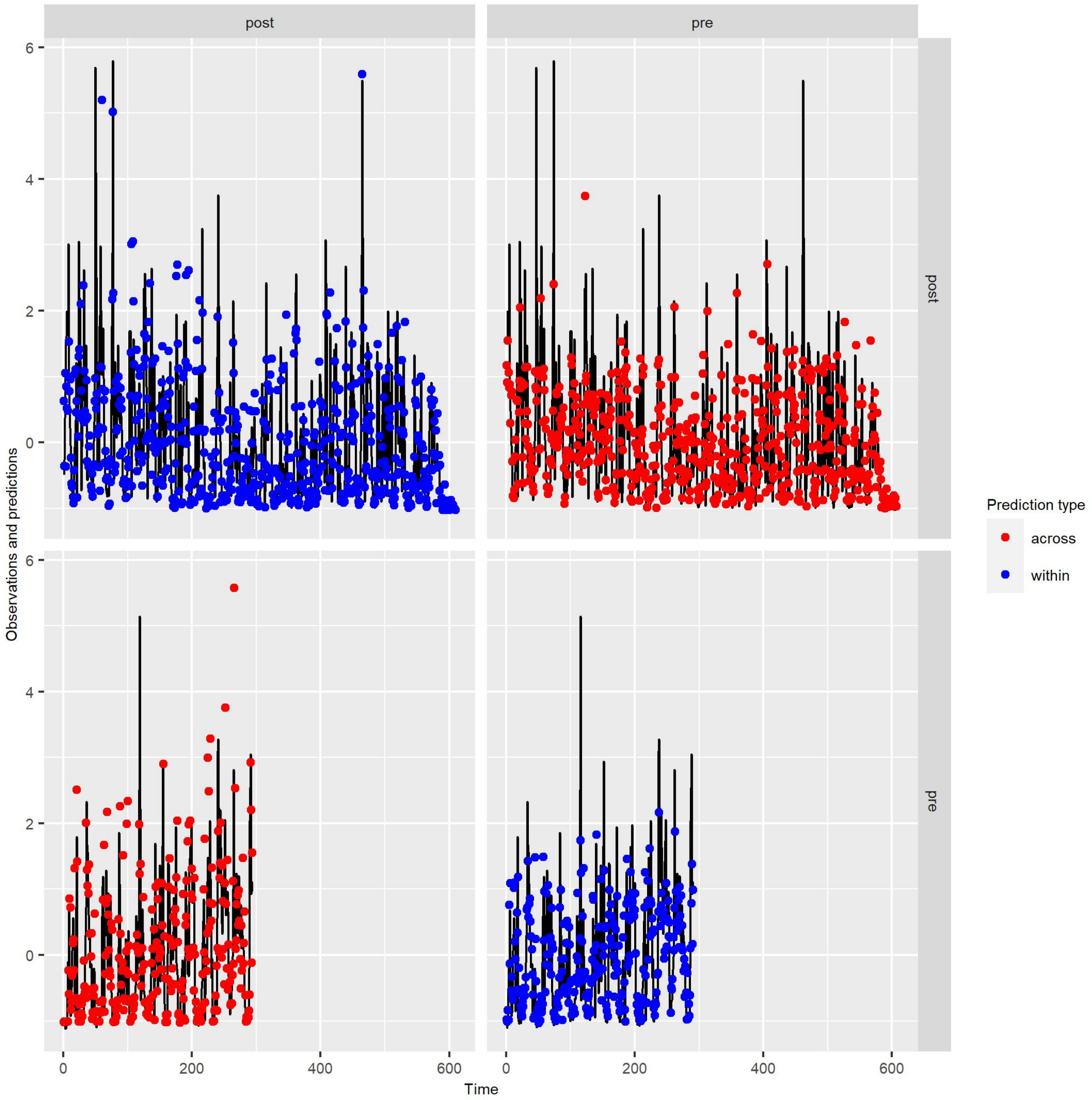

Supplemenentary Figure 12. Within and across regime predictions for a phytoplankton time series from Lake Müggelsee[S4]. Observed data is displayed as black lines. Across and within regime predictions are shown as red and blue dots, respectively. Columns show predictor regimes and rows display the observed regimes which are predicted. Pre and post refers to pre- and post-transition dynamics, respectively (See Fig. 5). The univariate prediction algorithm is here used to do predictions (See Methods).

Observed dynamics	Predictors	$MAPE_W$	$MAPE_A$
A	A	0.93	0.93
A	B	0.93	1.2
A	C	0.93	1
A	D	0.92	0.99
B	A	0.14	0.98
B	B	0.14	0.14
B	C	0.14	0.24
B	D	0.14	0.46
C	A	0.15	0.94
C	B	0.14	0.29
C	C	0.14	0.13
C	D	0.14	0.34
D	A	0.2	0.93
D	B	0.2	0.59
D	C	0.2	0.41
D	D	0.2	0.17

Supplementary Table 1. Mean absolute prediction errors for across and within regime predictions in data from the simulated food-chain model (Fig. 2, Material and Methods). Observed dynamics refers to the regime which is predict using information from another regime (Predictors). The observed dynamics is predicted using information both from the same regime and from another regime (Predictors), giving rise to different mean absolute prediction errors for within ($MAPE_W$; $\frac{1}{n}\sum_{t=1:n} \mathrm{abs}\left(\hat{Y}_{\boldsymbol{M}_A}(t)|\boldsymbol{M}_A - Y_{\boldsymbol{M}_A}(t)\right)$) and across ($MAPE_A$; $\frac{1}{n}\sum_{t=1:n} \mathrm{abs}\left(\hat{Y}_{\boldsymbol{M}_A}(t)|\boldsymbol{M}_B - Y_{\boldsymbol{M}_A}(t)\right)$) regime predictions, respectively. Indexes A, B, C and D refer to stochastic equilibrium, 2-point limit cycles, 4-point limit cycles and chaotic dynamics, respectively. Time series of the predator in the trophic chain model is here used to predict the other species′ dynamics. Mean absolute prediction errors are here shown for the case where time series length is 100 and the level of observation error 0.15.

Supplementary Discussion

Cross-validation vs. out-of-sample forecasts

It is recommended that a subset of a time series should be set aside for out-of-sample forecasts using attractor reconstruction techniques [S4]. However, here we have instead used a leave-one-out cross validation procedure[S5]. The primary reason for this is that long enough time series are seldom available in natural systems. Therefore, leave-one-out cross validations rather than out-of-sample forecasts, have often been used in applied studies using these techniques (e.g. [S5]). The second reason making a leave-one-out cross validation approach preferable to an out-of-sample forecast approach is that the data set used as a library to make forecasts should cover an as large part of the state space of a dynamical regime as possible. If only a subset of the state-space is covered in the library data set, predictions will be biased; an issue which can be illustrated through an example. First, consider a time series for which the power spectrum is dominated by low frequencies (i.e. long term cyclicity dominates the time series). If this time series is divided in two parts - a library and an out-of-sample data set - then the two parts may cover different portions of the whole data set due to the dominance of low frequencies (the library data set may, for example, cover the high values of the time series and the prediction data set the lower values of the time series). For the simplex algorithm, this yields biased predictions for the part of state space in the out-of-sample data set which is not a subset of the library data set. Thus, before applying our approach one should first investigate if library and prediction data sets cover different parts of state space.

If a univariate approach is used to detect alternative attractors then one would ideally have access to a long time series. Thereafter, for a given dynamical regime, the time series would be split in two parts: a library and a prediction data set. The embedding dimension E (the only parameter that is estimated using these techniques) would thereafter be estimated using a leave-one-out cross validation approach within the library data set for a given regime, and a "true" out-of-sample forecast would thereafter be conducted for both within and across regime predictions. This approach would imply that neither across nor within regime predictions were optimized for finding the models embedding dimension. Hence, prediction accuracy of neither within nor across regime predictions would be optimized in the estimation procedure.

However, for the multivariate approach, the aforementioned issue is not a problem. When the multivariate approach is used, another time series than the one involved in attractor reconstruction, is predicted. This means that both within and across regime forecasts are made on data that was not used to optimize the models embedding dimension.

Applications to field data

We have here developed an approach for testing if the temporal dynamics of ecosystems are different pre and post critical transitions. Before applying the approach, a first step is to decide which parts of a time series that constitute pre and post transition dynamics. This can either be done using biological knowledge of the specific system, or through formal statistical tests such as break-point analyses (See e.g. [S6]). Thereafter, as with any statistical hypothesis

test, the probability of detecting significant difference depends on the quality and amount data available, here specifically brought about as observation error and time series length (Fig. 2). To this end, these techniques have been shown to be relatively robust to observation errors[S7], yet long time series are required for efficient attractor reconstructions. A rule of thumb is that approximately 30 time steps are required[S7], but a sufficient time series length is context dependent and likely depends on sampling frequency in conjunction with the generation time of the investigated species[S5]. Our approach, indeed empirical dynamic modeling in general, is thus mainly applicable for frequently sampled short lived species or long time series of species with a long generation time. Nevertheless, it should be noted that attractor reconstructions can also be built by thoughtfully seeming together time series from spatial replicates[S8] or dynamically/ecologically similar species[S9], making the approach potentially applicable also for shorter time series.

Moreover, before applying the multivariate method of testing if alternative regimes are dynamically dissimilar one should first test if variables are dynamically coupled. This can be done using convergence cross-mapping[S7].

Supplementary References

S1. W. Govaerts, Y. Kuznetsov, H. Meijer, B. Al-Hdajbat, W. De Witte, A. Dhooge, W. Mestrom, N. Neirynck, A. Riet, B. Sautois. "MATCONT: continuation toolbox for ODEs in Matlab" https://sourceforge.net/projects/matcont/files/matcont/matcont6p11/

S2. Scheffer, M. *et al.* Early-warning signals for critical transitions. *Nature* **461**, 53–59 (2009).

S3. May, R. M. Thresholds and breakpoints in ecosystems with a multiplicity of stable states. *Nature* **269**, 471–477 (1977).

S4. Chang, C.-W., Ushio, M. & Hsieh, C. Empirical dynamic modeling for beginners. *Ecol Res* **32**, 785–796 (2017).

S5. Munch, S. B., Giron-Nava, A. & Sugihara, G. Nonlinear dynamics and noise in fisheries recruitment: A global meta-analysis. *Fish Fish* **19**, 964–973 (2018).

S6. Gsell, A. S. *et al.* Evaluating early-warning indicators of critical transitions in natural aquatic ecosystems. *Proc Natl Acad Sci USA* **113**, E8089–E8095 (2016).

S7. Sugihara, G. *et al.* Detecting Causality in Complex Ecosystems. *Science* **338**, 496–500 (2012).

S8. Clark, A. T. *et al.* Spatial convergent cross mapping to detect causal relationships from short time series. *Ecology* **96**, 1174–1181 (2015).

S9. Hsieh, C., Anderson, C., Sugihara, G., Bolker, A. E. B. M. & DeAngelis, E. D. L. Extending Nonlinear Analysis to Short Ecological Time Series. *The American Naturalist* **171**, 71–80 (2008).
