# Supplementary material for: Detecting alternative attractors in ecosystem dynamics
Source: Commun Biol. 2021 Aug 17;4:975. doi: 10.1038/s42003-021-02471-w (PMC8370982; doi:10.1038/s42003-021-02471-w)
Supplement: Supplementary file 3 — Reporting Summary [file 42003_2021_2471_MOESM3_ESM.pdf]

nature research

Corresponding author(s): Torbjörn Säterberg

Last updated by author(s): Jun 15, 2021

Reporting Summary

Nature Research wishes to improve the reproducibility of the work that we publish. This form provides structure for consistency and transparency in reporting. For further information on Nature Research policies, see our Editorial Policies and the Editorial Policy Checklist.

Statistics

For all statistical analyses, confirm that the following items are present in the figure legend, table legend, main text, or Methods section.

n/a	Confirmed	
☐	☒	The exact sample size (n) for each experimental group/condition, given as a discrete number and unit of measurement
☐	☒	A statement on whether measurements were taken from distinct samples or whether the same sample was measured repeatedly
☐	☒	The statistical test(s) used AND whether they are one- or two-sided<br>*Only common tests should be described solely by name; describe more complex techniques in the Methods section.*
☒	☐	A description of all covariates tested
☒	☐	A description of any assumptions or corrections, such as tests of normality and adjustment for multiple comparisons
☒	☐	A full description of the statistical parameters including central tendency (e.g. means) or other basic estimates (e.g. regression coefficient) AND variation (e.g. standard deviation) or associated estimates of uncertainty (e.g. confidence intervals)
☐	☒	For null hypothesis testing, the test statistic (e.g. F, t, r) with confidence intervals, effect sizes, degrees of freedom and P value noted<br>*Give P values as exact values whenever suitable.*
☒	☐	For Bayesian analysis, information on the choice of priors and Markov chain Monte Carlo settings
☒	☐	For hierarchical and complex designs, identification of the appropriate level for tests and full reporting of outcomes
☒	☐	Estimates of effect sizes (e.g. Cohen's d, Pearson's r), indicating how they were calculated

Our web collection on statistics for biologists contains articles on many of the points above.

Software and code

Policy information about availability of computer code

Data collection	The simulated data, which was used to test the methodology described in this study, was simulated using different packages in matlab (R2020a) and R(version 4.0.1). Matcont(version 6.11; Supplementary reference #1) was used for bifurcation analyses, the SDE-tools (version 1.2; reference #35) was used to integrate stochastic differential equations and the deSolve-package (reference #29) was used to integrate the food-chain model.
Data analysis	Within and across regime predictions were calculated using empirical dynamic modeling. These algorithms are described in Sugihara & May (Nature 1990) and Sugihara et al. (Science 2012). For univariate predictions we used the R-package rEDM(referens #30) and for multivariate predictions(CCM-predictions) we wrote custome code in R(version 4.0.1) since the CCM method in the rEDM-package does not give predictions as output.<br><br>The simple non-parameteric bootstrap test, which was used to test if prediction errors of within and across regime predictions are significant different, was conducted using custome code in R (version 4.0.1).

For manuscripts utilizing custom algorithms or software that are central to the research but not yet described in published literature, software must be made available to editors and reviewers. We strongly encourage code deposition in a community repository (e.g. GitHub). See the Nature Research guidelines for submitting code & software for further information.

Data

Policy information about availability of data

All manuscripts must include a data availability statement. This statement should provide the following information, where applicable:

- Accession codes, unique identifiers, or web links for publicly available datasets
- A list of figures that have associated raw data
- A description of any restrictions on data availability

The experimental data set and the phytoplankton time series analyzed in this study were gathered from two previous studies (ref 7 & 19) and all other data was simulated using theoretical models. All figures have associated raw data, which is available through two open repositories (ref 37 & 38).

Field-specific reporting

Please select the one below that is the best fit for your research. If you are not sure, read the appropriate sections before making your selection.

☐ Life sciences ☐ Behavioural & social sciences ☒ Ecological, evolutionary & environmental sciences

For a reference copy of the document with all sections, see nature.com/documents/nr-reporting-summary-flat.pdf

Ecological, evolutionary & environmental sciences study design

All studies must disclose on these points even when the disclosure is negative.

Study description	This study introduces a novel time series analysis approach for testing ecosystems exhibit alternative dynamical attractors.
Research sample	The methodology developed in this study was tested using simulated, experimental and natural time series data. The simulated data was derived using theoretical model. The experimental data was produced from predator-prey chemostat experiments in a previous study by Fussman et al. (Science 2000). This study found that a Hopf-bifurcation was apparent in the system, yet they did not specifically test if the time series´ were temporarily different. The natural time series data was witheld from a previous study investigating early warning signals in aquatic ecosystems (Gsell et al. 2016).
Sampling strategy	This paper introduces a new method where we, as a part of the analysis, test the data requirements of the method.
Data collection	This study uses already collected (Fussman et al. 2000 Science; Gsell et al. 2016 PNAS) and simulated data
Timing and spatial scale	No data was collected during this study.
Data exclusions	No data was excluded
Reproducibility	Code and data for reproducing this study is avaliable at : http://doi.org/10.5281/zenodo.4953854
Randomization	Not apropriate here since we develop a novel time series analysis approach in this paper.
Blinding	Manipulative experiments were not conducted in this study. Blinding is thus not relevant

Did the study involve field work? ☐ Yes ☒ No

Reporting for specific materials, systems and methods

We require information from authors about some types of materials, experimental systems and methods used in many studies. Here, indicate whether each material, system or method listed is relevant to your study. If you are not sure if a list item applies to your research, read the appropriate section before selecting a response.

Materials & experimental systems

n/a	Involved in the study	
☒	☐	Antibodies
☒	☐	Eukaryotic cell lines
☒	☐	Palaeontology and archaeology
☒	☐	Animals and other organisms
☒	☐	Human research participants
☒	☐	Clinical data
☒	☐	Dual use research of concern

Methods

n/a	Involved in the study	
☒	☐	ChIP-seq
☒	☐	Flow cytometry
☒	☐	MRI-based neuroimaging
